# Risky health behaviors during the COVID-19 pandemic: Evidence from the expenditures on alcohol, non-alcoholic beverages, and tobacco products

**DOI:** 10.1371/journal.pone.0268068

**Published:** 2022-05-19

**Authors:** Binod Acharya, Chandra Dhakal

**Affiliations:** 1 Urban Health Collaborative, Drexel University, Philadelphia, Pennsylvania, United States of America; 2 Department of Agricultural and Applied Economics, University of Georgia, Athens, Georgia, United States of America; IZA - Institute of Labor Economics, GERMANY

## Abstract

**Background:**

The COVID-19 pandemic has increased mental stress among the population and, at the same time, has lowered consumer income. Alcohol, non-alcoholic beverages, and tobacco consumption are associated with multiple health conditions but the information on how the consumption pattern of these goods shifted during the pandemic remains limited.

**Objective:**

To examine the consumer spending on alcohol, non-alcoholic beverages, and tobacco products during the COVID-19 pandemic compared to the pre-pandemic period.

**Design:**

An observational study utilizing the expenditures data on alcohol, non-alcoholic beverages, and tobacco between 2017 and 2020 obtained from the US Consumer Expenditure Diary Survey.

**Participants:**

18,808 respondents aged ≥ 21 years who answered the Consumer Expenditure Diary Survey.

Main Outcome Measure(s): Bi-weekly expenditure on alcohol, non-alcoholic beverages, and tobacco products.

**Analysis:**

Multivariable linear regression models.

**Results:**

A total of 18,808 respondents (mean [SD] age = 52.5[16.9] years; 53.8% females) were included. Compared to the pre-pandemic levels, household expenditures on alcohol, non-alcoholic beverages, and tobacco products significantly decreased during the pandemic period by 28.6%, 7.9%, and 15.5%, respectively, after controlling for the state-, individual-, and household-level characteristics. Individual age, race/ethnicity, income, and education were significant predictors of spending. Heterogeneities in expenditures were evident across subgroups, with less educated and low-income households cutting their alcohol expenses while the wealthy and more educated consumers spent more during the pandemic.

**Conclusions and implications:**

Household expenditures on alcohol, non-alcoholic beverages, and tobacco products significantly decreased. The results might be beneficial in understanding consumer spending habits concerning risky health behaviors during the period of economic disruption.

## Introduction

The COVID-19 pandemic has created unprecedented disruptions in public health and the economies around the globe. Apart from the enormous death tolls and hospitalizations, the pandemic and the subsequent public health interventions such as stay-at-home orders, lockdowns, and business closures have created massive unemployment rates, foreclosures, and layoffs. In the United States, the national unemployment rate hit 14.7% in April 2020 and remained at more than 7% for six consecutive months [1]. Because of the pandemic, consumer spending in many major categories decreased, industrial production declined, and the economy entered a recession [2]. The pandemic also exerted an enormous impact on mental health, as documented by multiple studies reporting increased psychological distress, anxiety, depression, insomnia, and suicidal ideation [3–6].

Studies on alcoholic and non-alcoholic beverage spending and smoking habits are important for epidemiological and public health reasons. High alcohol consumption is associated with many health impairments, including liver diseases, cancer, injuries and accidents, and mental well-being [7–10]. Non-alcoholic sugar-sweetened beverages are implicated as the risk factors for morbidities like diabetes and obesity [11–13]. Similarly, smoking is a well-known risk factor for multiple diseases, including respiratory diseases, cardiovascular diseases, and cancer. Consumption of alcohol has increased in the U.S. over the past years [14], and there are fears that the pandemic exacerbates this trend [15, 16]. Although the longer-term smoking rate has declined in the U.S., the smoking prevalence among young adults remains high [17]. It is likely that the financial strain and physical and mental health crisis brought about by the pandemic could affect people’s lifestyles, including their food, drinking, and smoking habits. Because of the public-health consequences of alcohol use, smoking, and non-alcoholic beverage consumption, examining possible shifts in these habits during the pandemic is important in adopting appropriate public health measures and allocating public funds.

Past studies have reported that the consumption of alcohol and other drugs usage increases at the time of mental and psychological stress. For instance, higher alcohol consumption has been reported after the 9/11 terrorist attack [18] and after Hurricane Katrina [19]. Although the social and economic context of the current pandemic could be very much different than those stressful events, this evidence nonetheless appears to support the hypothesis that people tend to use alcohol for self-medication, a kind of coping strategy for mental and emotional distress [20–25]. Given that the COVID-19 pandemic has impacted mental health on an enormous scale, the “coping” hypothesis would predict a higher alcohol consumption after the onset of the pandemic. On the other hand, there are reasons that alcohol consumption might decrease during the pandemic since social gatherings and sporting events were prohibited, and pubs and clubs were closed during the lockdown. Furthermore, the high unemployment rates, wage cuts, and financial hardship during the pandemic might have reduced consumer income, forcing people to cut their alcohol, tobacco, or other beverage consumption. This “income-effect” hypothesis has been widely studied in relation to alcohol consumption during the past macroeconomic shocks. Most studies have reported a positive association between economic decline, often proxied by the unemployment rate, and reduced alcohol consumption [26–28]. Using Nielsen Homescan data, similar pro-cyclical behavior of alcohol sales has been reported by Cotti et al. [29]. A few other studies report the counter-cyclical behavior of alcohol consumption [30, 31]. The mixed results on the behavior of alcohol consumption [32] during economic decline remains a subject of the ongoing inquiry, but they might arise due to differences in the level of analysis (e.g. individual level versus state level), a measure of alcohol consumption (e.g. frequency of consumption versus dollars spent), the data sources or the analytic framework used. A recent 2017 study by Carpenter et al. [33] found that clinically relevant alcohol use disorder was positively associated with unemployment rates even though the total alcohol consumption was not, highlighting the importance of the metric chosen to measure alcohol consumption. The consumption habits may also be different across sub-populations. In fact, the rise in abstention in alcohol consumption among light drinkers but the increase in total consumption due to binge drinking by heavy drinkers during the Great Recession of 2008–2009 lends some evidence of differential alcohol consumption habits among sub-populations [34].

It is plausible in the case of the COVID-19 pandemic that both types of forces–those that help to cut alcohol consumption and those that exacerbate alcohol use–are in play but in the opposite direction. It, therefore, is an empirical question, using reliable data, to determine whether alcohol consumption increased during the pandemic. Some studies outside the U.S. indicate a higher alcohol consumption during the pandemic [35, 36]. Only a few studies have examined the trend of alcohol sales and consumption in the U.S. during the pandemic and report a higher alcohol consumption and sales, but these studies either do not account for the macroeconomic characteristics or rely on a smaller survey sample over a narrow study window [37–39]. The studies on the impact of the COVID-19 pandemic on non-alcoholic beverages consumption and smoking are conspicuously lacking. This study examines the changes in consumer spending on alcohol, non-alcoholic beverages, and tobacco products by exploiting the pandemic-enabled natural experiment. Furthermore, we hypothesize that the pandemic may affect expenditure on alcohol, non-alcoholic beverages, and tobacco products among some individuals more than others. Recognizing the possibility of heterogeneous spending habits among different socio-economic groups, we will perform subgroup analyses by gender, race/ethnicity, education, and income levels.

## Methods

### Study setting and data

This is a retrospective observational study that uses data from January 2017 to December 2020. We obtained the public use microdata (PUMD) of expenditure on alcohol, non-alcoholic beverages, and tobacco products for on- or off-premise use from the Consumer Expenditure Survey (CEX) Diary Survey, conducted by the U.S. Census Bureau. The CEX collects information on frequently purchased items via the Diary Survey, a cross-sectional survey requiring households to report one-week expenditures for two consecutive weeks. The survey design involves a two-step process in which a random sample of geographic areas is selected, and then a random sample of households is selected inside those areas. The geographic areas are small clusters of counties, called primary sampling units. The survey is designed to produce unbiased expenditure estimates at the national, Census Region, and Census Division levels, but not at the state or counties level. The average response rates were 58%, 55%, 53%, 35% for survey years 2017, 2018, 2019, and 2020, respectively [40, 41]. The methodology of the survey is discussed elsewhere [42]. The alcohol products comprise beer and ale, whiskey, wine, or other alcoholic beverages. The non-alcoholic beverages include Cola, other carbonated drinks, coffee, tea, non-carbonated fruit-flavored drinks, non-alcoholic beer, sports drinks, and other non-carbonated drinks. The tobacco products and smoking supplies include cigarettes, other tobacco products (including e-cigarettes), smoking accessories, and marijuana. We restricted the analysis to the purchases made by individuals aged at least 21 years, the minimum legal age for alcohol and tobacco purchase. We defined the period between March 2020 and December 2020 as the “pandemic period” and the prior time as the “pre-pandemic period.” We aggregated the data into a bi-weekly period and log-transformed after adding $0.5 in all three spending categories to handle the potential problem of very small or zero weekly purchases. To account for the inflation, the expenditures were adjusted to January 2017 price with their respective consumer price indices obtained from BLS [43]. The data on state-level monthly unemployment rates were also obtained from the U.S. Bureau of Labor Statistics (BLS).

### Statistical analysis

We separately performed multivariable linear regression analyses on bi-weekly log-transformed spending on alcohol, non-alcoholic beverages, and tobacco products. Our base model includes the indicator variable denoting whether the expenditure is made during the pandemic or pre-pandemic periods as the major explanatory variable of interest. We also included the state-level monthly unemployment rate as a covariate, a proxy for the macro-economic conditions to control for its potential impact on spending. The other individual-level covariables were individual age (4 categories: 21–35 years, 36–50 years, 51–65 years, > 65 years), gender (2 categories), race/ethnicity (4 categories: Non-Hispanic White, Non-Hispanic Black, Hispanic, Other), annual household income in the past 12 months (4 categories: < $25000, $25000–50000, $50000–100000, >$100000), and the highest level of education (3 categories: < high school, high school, college including an associate degree from college). Household characteristics included were the indicator variable denoting whether or not any household member received food stamps in the past 12 months and family size (numerical). We also included fixed effect terms for months of the expenditure, the Census-defined region where the respondent lived (4 categories: West, Midwest, South, Northeast), and the indicator variable denoting urban/rural home address. The analysis was weighted by the survey weights provided by the CEX. We ran four additional models to examine the differential expenditure pattern across different socio-economic groups for each outcome (expenditure on alcohol, non-alcoholic beverages, and tobacco products). These models included the covariates in the base model plus an interaction term between the indicator for pandemic and gender, race/ethnicity, income, and education, one at a time. This allowed us to quantify differential impacts of the pandemic in our outcomes of interest across a dimension of household characteristics such as gender, race/ethnicity, education, and income levels using the linear combination of the adjusted regression coefficients. The statistical analysis was carried out in R version 4.0.2.

## Results

The demographic and socio-economic characteristics of the study sample (unweighted, N = 18,808) are presented in Table 1. There were a relatively similar number of respondents and had fairly similar characteristics across the survey years. Less than 8% of the overall sample had education below high school, whereas more than 52% had a minimum college-level education. About 41% of the respondents had an annual household income below $50,000.

**Table 1:** Demographic and socio-economic characteristics of the study sample (unweighted).

Characteristics	No. (%)				
	All years (N = 18,808)	2017 (N = 4889)	2018 (N = 4659)	2019 (N = 4524)	2020 (N = 4736)
Age (years)					
21 to 35	3748 (19.9)	1033 (21.1)	944 (20.3)	920 (20.3)	851 (18.0)
36 to 50	4867 (25.9)	1243 (25.4)	1238 (26.6)	1177 (26.0)	1209 (25.5)
51 to 65	5506 (29.3)	1438 (29.4)	1373 (29.5)	1318 (29.1)	1377 (29.1)
65 plus	4687 (24.9)	1175 (24.0)	1104 (23.7)	1109 (24.5)	1299 (27.4)
Female	10114 (53.8)	2658 (54.4)	2512 (53.9)	2409 (53.2)	2535 (53.5)
Race/Ethnicity					
Non-Hispanic White	12823 (68.2)	3326 (68.0)	3181 (68.3)	3089 (68.3)	3227 (68.1)
Non-Hispanic Black	1851 (9.8)	506 (10.3)	452 (9.7)	432 (9.5)	461 (9.7)
Hispanic	2512 (13.4)	664 (13.6)	647 (13.9)	621 (13.7)	580 (12.2)
Other	1622 (8.6)	393 (8.0)	379 (8.1)	382 (8.4)	468 (9.9)
Food stamps in the past 12 months					
Received	1496 (8.2)	436 (9.2)	367 (8.2)	321 (7.4)	372 (8.1)
Not received	16661 (91.8)	4285 (90.8)	4120 (91.8)	4045 (92.6)	4211 (91.9)
Education					
Less than high school	1476 (7.8)	410 (8.4)	381 (8.2)	380 (8.4)	305 (6.4)
High school	7539 (40.1)	2031 (41.5)	1914 (41.1)	1804 (39.9)	1790 (37.8)
College	9793 (52.1)	2448 (50.1)	2364 (50.7)	2340 (51.7)	2641 (55.8)
Annual household income					
<$25000	3543 (18.9)	1003 (20.5)	912 (19.6)	816 (18.1)	812 (17.2)
$25000-$50000	4136 (22.0)	1132 (23.2)	1028 (22.1)	992 (22.0)	984 (20.8)
$50000-$100000	5347 (28.5)	1389 (28.4)	1350 (29.0)	1308 (29.0)	1300 (27.5)
>$100000	5753 (30.6)	1359 (27.8)	1365 (29.3)	1402 (31.0)	1627 (34.4)
Census region					
Midwest	3759 (20.0)	986 (20.2)	884 (19.0)	894 (19.8)	995 (21.0)
Northeast	3323 (17.7)	935 (19.1)	868 (18.6)	758 (16.8)	762 (16.1)
South	6411 (34.1)	1764 (36.1)	1637 (35.1)	1552 (34.3)	1458 (30.8)
West	5315 (28.3)	1204 (24.6)	1270 (27.3)	1320 (29.2)	1521 (32.1)

**Fig 1:**
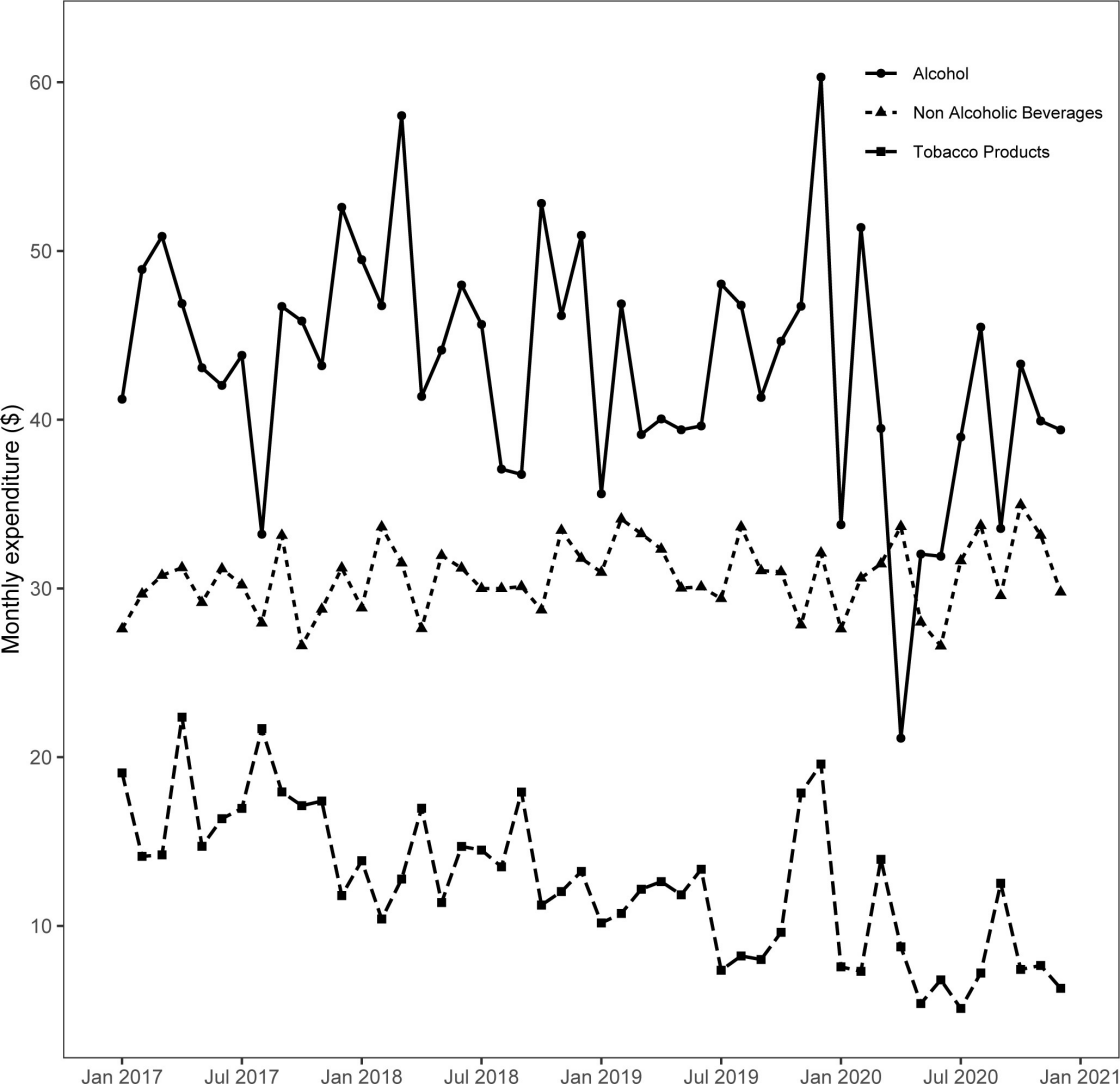
The trend of monthly household expenditure dollars on alcohol, non-alcoholic beverages, and tobacco products.

Fig 1 shows raw trends of the weighted monthly expenditure by a typical U.S. household on alcohol, non-alcoholic beverages, and tobacco products. The household expenditure on alcohol is considerably higher than that on non-alcoholic beverages or tobacco products. The average monthly expenditure on alcohol in the pre-pandemic period was $45.0 versus the pandemic period average of $36.5. The mean monthly spending on non-alcoholic beverages was similar between the pre-pandemic ($30.6) and the pandemic period ($31.1), but in the case of tobacco products, pandemic period expenditure ($8.1) was notably smaller than the pre-pandemic expenditure ($13.8).

We present the regression coefficients of multiple linear regression analyses from our base models in Table 2. Since we modeled the log(expenditure) as the outcome variable, we can estimate that a unit change (or compared to the reference group in the case of categorical predictor) in the predictor variable is associated with $(e^{\hat{\beta }}-1)\times 100\%$ change in expenditure. The dummy variable denoting the pandemic versus pre-pandemic was significantly associated with the bi-weekly spending on alcohol, non-alcoholic beverage, or tobacco products. The association was much more prominent in the alcohol expenditure (estimate = -0.337, 95% CI: -0.409; -0.264), which corresponds to a decline of 28.6% (95% CI: 23.2%; 33.6%) during the pandemic period compared to the pre-pandemic period. The expenditure on tobacco products decreased by 15.5% (95% CI: 11.31%; 19.3%) during the pandemic period. In the case of non-alcoholic beverages, we report a modest decline of 7.9% (95% CI: 2.2%; 13.3%). The state-level monthly unemployment rate was not significantly associated with the expenditure in all three expenditure categories. In general, the demographic and socio-economic characteristics were statistically significant across all three expenditure categories.

**Table 2:** Estimated regression coefficients from the base models for spending on alcohol, non-alcoholic beverages, and tobacco products.

Variable	Alcohol		Non-alcoholic beverages		Tobacco products	
	β [95% CI]	p-value	β [95% CI]	p-value	β [95% CI]	p-value
Pandemic (Ref: Pre-pandemic)	-0.337[-0.409, -0.264]	<0.001	-0.082[-0.143, -0.022]	0.008	-0.168[-0.215, -0.12]	<0.001
Unemployment rate	0.008[-0.002, 0.019]	0.111	0.007[-0.001, 0.016]	0.088	0.007[-0.001, 0.014]	0.088
Age group (Ref: 21 to 35)						
36 to 50 years	-0.094[-0.176, -0.011]	0.026	0.272[0.207, 0.338]	<0.001	0.11[0.052, 0.169]	<0.001
51 to 64 years	-0.092[-0.173, -0.011]	0.026	0.364[0.3, 0.428]	<0.001	0.048[-0.009, 0.104]	0.098
65 years and above	-0.271[-0.355, -0.187]	<0.001	0.211[0.144, 0.278]	<0.001	-0.269[-0.322, -0.216]	<0.001
Annual household income (Ref: < $25,000)						
$25000-$50000	0.311[0.237, 0.385]	<0.001	0.154[0.086, 0.223]	<0.001	-0.005[-0.066, 0.057]	0.883
$50000-$100000	0.569[0.489, 0.649]	<0.001	0.238[0.168, 0.308]	<0.001	-0.01[-0.073, 0.054]	0.766
>$100000	1.147[1.054, 1.239]	<0.001	0.399[0.322, 0.476]	<0.001	-0.071[-0.14, -0.002]	0.045
Gender (Female: Ref: Male)	-0.168[-0.223, -0.112]	<0.001	0.002[-0.041, 0.046]	0.912	-0.121[-0.159, -0.083]	<0.001
Education (Ref: < High school)						
High school	0.121[0.032, 0.211]	0.008	-0.016[-0.103, 0.071]	0.721	0.024[-0.056, 0.105]	0.552
College	0.404[0.31, 0.499]	<0.001	0.002[-0.087, 0.092]	0.958	-0.216[-0.298, -0.135]	<0.001
Race/Ethnicity (Ref: Hispanic)						
Non-Hispanic Black	-0.273[-0.376, -0.169]	<0.001	-0.216[-0.311, -0.121]	<0.001	0.126[0.051, 0.202]	0.001
Non-Hispanic White	0.232[0.152, 0.312]	<0.001	0.217[0.148, 0.287]	<0.001	0.347[0.289, 0.404]	<0.001
Other	-0.332[-0.449, -0.216]	<0.001	-0.177[-0.276, -0.078]	<0.001	0.178[0.101, 0.255]	<0.001
Month (Ref: Jan)						
February	0.064[-0.064, 0.191]	0.328	0.052[-0.048, 0.152]	0.311	-0.064[-0.151, 0.023]	0.148
March	0.044[-0.08, 0.169]	0.485	0.085[-0.016, 0.186]	0.099	0.024[-0.064, 0.113]	0.589
April	0.043[-0.09, 0.176]	0.527	0.079[-0.028, 0.186]	0.146	0.108[0.011, 0.206]	0.030
May	0.04[-0.09, 0.171]	0.545	0.016[-0.089, 0.121]	0.762	-0.007[-0.096, 0.082]	0.876
June	-0.005[-0.141, 0.13]	0.939	0.012[-0.093, 0.118]	0.820	0.04[-0.058, 0.138]	0.420
July	0.167[0.03, 0.304]	0.017	-0.033[-0.139, 0.073]	0.546	0.005[-0.087, 0.096]	0.920
August	0.014[-0.113, 0.141]	0.829	0.023[-0.081, 0.127]	0.664	0.073[-0.018, 0.165]	0.116
September	0.01[-0.12, 0.14]	0.879	0.064[-0.038, 0.165]	0.219	0.006[-0.086, 0.098]	0.898
October	0.151[0.024, 0.277]	0.020	0.014[-0.087, 0.114]	0.793	0.027[-0.063, 0.116]	0.559
November	0.114[-0.016, 0.244]	0.086	0.081[-0.022, 0.183]	0.124	0.096[0.002, 0.191]	0.045
December	0.097[-0.036, 0.231]	0.152	0.041[-0.064, 0.145]	0.446	0.008[-0.084, 0.101]	0.862
Food-stamps received (Ref: Not received)	0.099[0.015, 0.183]	0.021	0.033[-0.053, 0.119]	0.447	-0.094[-0.171, -0.017]	0.017
Family size, No.	-0.119[-0.141, -0.098]	<0.001	0.175[0.156, 0.193]	<0.001	0.008[-0.008, 0.024]	0.322
Census region (Ref: Midwest)						
Northeast	-0.092[-0.179, -0.004]	0.041	-0.007[-0.074, 0.061]	0.847	-0.098[-0.16, -0.035]	0.002
South	-0.129[-0.204, -0.053]	<0.001	-0.002[-0.062, 0.058]	0.949	-0.051[-0.105, 0.004]	0.067
West	0.032[-0.05, 0.113]	0.444	-0.071[-0.134, -0.007]	0.030	-0.129[-0.185, -0.073]	<0.001
Urban (Ref: Rural)	0.406[0.239, 0.574]	<0.001	-0.252[-0.42, -0.085]	0.003	-0.154[-0.339, 0.03]	0.101
Intercept	0.288[0.013, 0.563]	0.040	1.129[0.858, 1.400]	<0.001	0.747[0.478, 1.015]	<0.001

**Fig 2:**
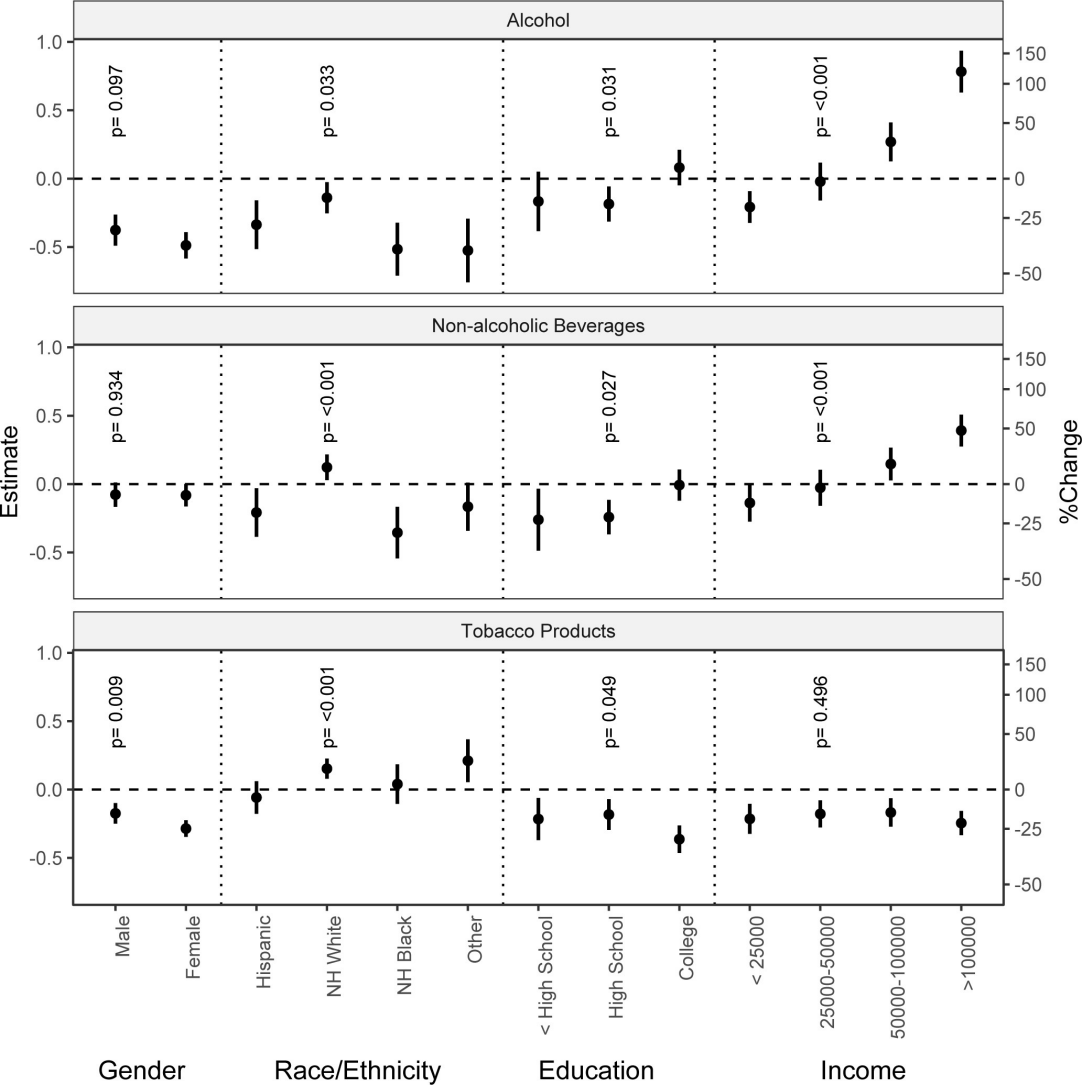
Changes in spending (estimate and 95% CI) by expenditure categories and socio-economic groups during the pandemic estimated from the interaction models. The p-values represent the test of similarity in expenditure in the pandemic period within categories of the given socio-economic group (gender, race/ethnicity, education, income).

Fig 2 presents the key results from the regression models that included the interaction term between the indicator variable of the pandemic period and socio-economic groups. We display the magnitude of changes in the expenditure in categories of the socio-demographic groups during the pandemic compared to the pre-pandemic levels. The pattern of changes in alcohol, non-alcoholic beverage, and smoking expenditure during the pandemic compared to pre-pandemic was similar between males and females. Both groups cut their alcohol and smoking expenditures (p = 0.097). A reduction in alcohol expenditure was seen across all race/ethnicity categories, but the drop among non-Hispanic Whites was smaller (13%) than non-Hispanic Blacks (40%). The expenditure on non-alcoholic beverages and tobacco products increased among non-Hispanic Whites. The spending on alcoholic and non-alcoholic beverages during the pandemic decreased among the lowest-income group (<$25,000), did not change among the middle-income group ($25,000-$50,000), and increased among two high-income groups. In the highest income group, the alcohol expenditure more than doubled during the pandemic compared to the pre-pandemic level. The spending on tobacco products decreased among all income groups in a similar fashion, ranging between 15% to 22%. The alcohol and non-alcoholic beverage expenditure decreased among those with high school or less education but not college-educated. The expenditure on tobacco products decreased across all education levels with about 17% reduction among high-school graduates but with a more pronounced effect of approximately 30% decline among college-educated.

## Discussion

Leveraging the nationally-representative CEX data, this study examined the changes in bi-weekly spending on alcohol, non-alcoholic beverages, and tobacco products attributable to the COVID-19 pandemic. After adjusting for other covariates, alcohol, non-alcoholic beverage, and tobacco spending during the pandemic significantly decreased by 28.6%, 7.9%, and 15.5%, respectively, from the pre-pandemic level.

Our results contrast with existing studies examining alcohol consumption or spending during the pandemic [37–39, 44]. These studies examine the changes in alcohol consumption or sales during a much shorter window, perhaps due to data limitation [38], making it harder to obtain a clear trend and understand the potential effects of the pandemic on alcohol consumption. For instance, McKetta et al. [44], using a subjective measure of the number of drinking days between March 10 to June 8, 2020, reported an increase in alcohol consumption. Pollard et al. [37] analyzed a small survey of 1,540 adults where they assessed alcohol consumption for only 20 days during the pandemic in a survey with a 9% response rate. Nordeck et al. [39] examined the changes in alcohol consumption between March 11 to July 21, 2020, without including the consumption pattern in the pre-pandemic period. In fact, Lee et al.’s [38] findings of an increase in alcohol sales in the three months between April to June 2020 are visibly similar to what we report in Fig 1, but when we extend our analysis to the end of 2020, the overall alcohol expenditure decreases. Our study overcomes the limitations of existing studies by (1) examining the expenditure data for a longer pandemic period, (2) including the pre-pandemic data for comparison, and (3) utilizing a long-running nationally representative survey that has a relatively high response rate. Our results largely agree with the BLS report that announced that the alcoholic beverages expenditures decreased by 17.4% in 2020 compared to the previous year [45]. Note that our estimates compared the changes during the pandemic (March-December, 2020) relative to January 2017-February 2020 adjusting for other covariates, which may explain the differences in estimated magnitudes between the BLS report and our results. Our analytic framework does not elucidate the reasons, but a combination of factors may have likely contributed to this decrease in expenditure. Closure or limited opening of restaurants and pubs and the people’s decision to avoid the crowded places might have diminished the on-premise alcohol consumption more than what could be offset by the increase in alcohol consumption at home [45]. Although the expenditure on tobacco products and non-alcoholic beverages also decreased during the pandemic, the magnitude of the decrease was smaller compared to the alcoholic beverage.

Unlike alcohol consumption which could be impacted heavily by closures of restaurants, pubs, social gatherings, or other on-premise consumption sites, non-alcoholic beverages consumption and smoking can typically occur at home. This might be a likely reason why we observed a considerable decline in alcohol expenditure but only a smaller decline in non-alcoholic beverages and smoking spending. Moreover, the financial strain, restaurant and bar closure, and the reduced disposable income during the pandemic might have forced consumers to cut their alcohol expenditure.

Past studies examining the alcohol consumption pattern during the macro-economic decline differ in their conclusion. The majority of studies find a pro-cyclical consumption pattern, with a decrease in alcohol intake as the economy declines [26–28, 34, 46]. The emerging evidence broadly implies that alcohol consumption decreased among individuals experiencing joblessness while the larger population reduced their intake [47]. To the extent the pandemic captures the economic hardship faced by the consumers, our findings appear to support these findings. Our findings imply that the reduction in expenditure due to the income-effect or avoidance of on-premise consumption prevails over the possible increase in alcohol use for coping with mental distress. To better understand the changes in alcohol consumption brought about by the pandemic, future studies focusing on the dynamics of on-premise and off-premise drinking accounting for the price differentials by place of consumption are warranted.

Although we saw a general decline in expenditure, particularly in alcohol and tobacco products, there were some notable heterogeneities across sub-groups. Compared to the pre-pandemic level, the expenditure on alcoholic and non-alcoholic beverages decreased among the low-income and less-educated groups. Past studies indicate that people in the higher-socioeconomic strata have more drinking days even though people in the lower socio-economic strata tend to have more frequent heavy drinking episodes and binge drinking [48–50]. It is likely that the disruptions of social gatherings and parties, where binge drinking is common, might have led to a decrease in alcohol consumption among people in lower socioeconomic strata. Moreover, the decline in alcohol expenditure among poor and less educated groups might partly explain the income effect since these groups are more vulnerable to financial strain during the pandemic.

## Limitations

We acknowledge several limitations of this paper. We could not account for the differences in expenditure by drink types or beverage groups. Spirits/distilled liquors are more expensive, and some non-alcoholic beverages are more costly than others. Consumers may be substituting expensive alcohols/drinks with relatively cheap beverages like beer, a drink of choice in the home [51, 52], especially when on-premise consumption is limited. We also note that there might be under-reporting expenditures in consumer surveys [50], particularly in alcohol and tobacco consumption, due to social desirability bias, even though the CEX is considered less prone to underreporting than the Homescan data [29]. Furthermore, we analyzed the expenditure but not the frequency or volume of alcohol, smoking, or non-alcoholic beverage consumption, which could be complementary measures of consumption behavior. Since the CEX samples were representative only at the national level, this study could not account for variation in pandemic-related policies by state and local governments (e.g. lockdowns) that might influence household expenditure decisions. While the CEX diary survey records the weekly expenses on frequently-purchased items, it does not capture less frequent large expenditures (e.g. mortgages), rendering the data on total household expenditure unavailable. This prevented us from modeling the share of expenditure on alcohol, tobacco products, and non-alcoholic beverages out of the total household expenditure. Lastly, we lacked individual-level employment information which could influence the expenditure pattern.

## Conclusions

In summary, we find that alcohol, non-alcoholic beverage, and tobacco expenditure decreased during the COVID-19 pandemic. The heterogeneous response across the demographic and socio-economic groups was evident. Individuals with less education and less income reduced their alcohol expenses, while the wealthy and educated consumers spent more during the pandemic. The findings from the study could be valuable for behavioral economists and public health professionals interested in examining food and drinking habits during periods of economic crisis and psychological distress.
